# Supplementary material for: An atlas of amyloid aggregation: the impact of substitutions, insertions, deletions and truncations on amyloid beta fibril nucleation
Source: Nat Commun. 2022 Nov 18;13:7084. doi: 10.1038/s41467-022-34742-3 (PMC9674652; doi:10.1038/s41467-022-34742-3)
Supplement: Supplementary file 1 — Supplementary information [file 41467_2022_34742_MOESM1_ESM.pdf]

Supplementary Information

An atlas of amyloid aggregation: the impact of substitutions, insertions, deletions and truncations on amyloid beta fibril nucleation

Seuma, Lehner & Bolognesi

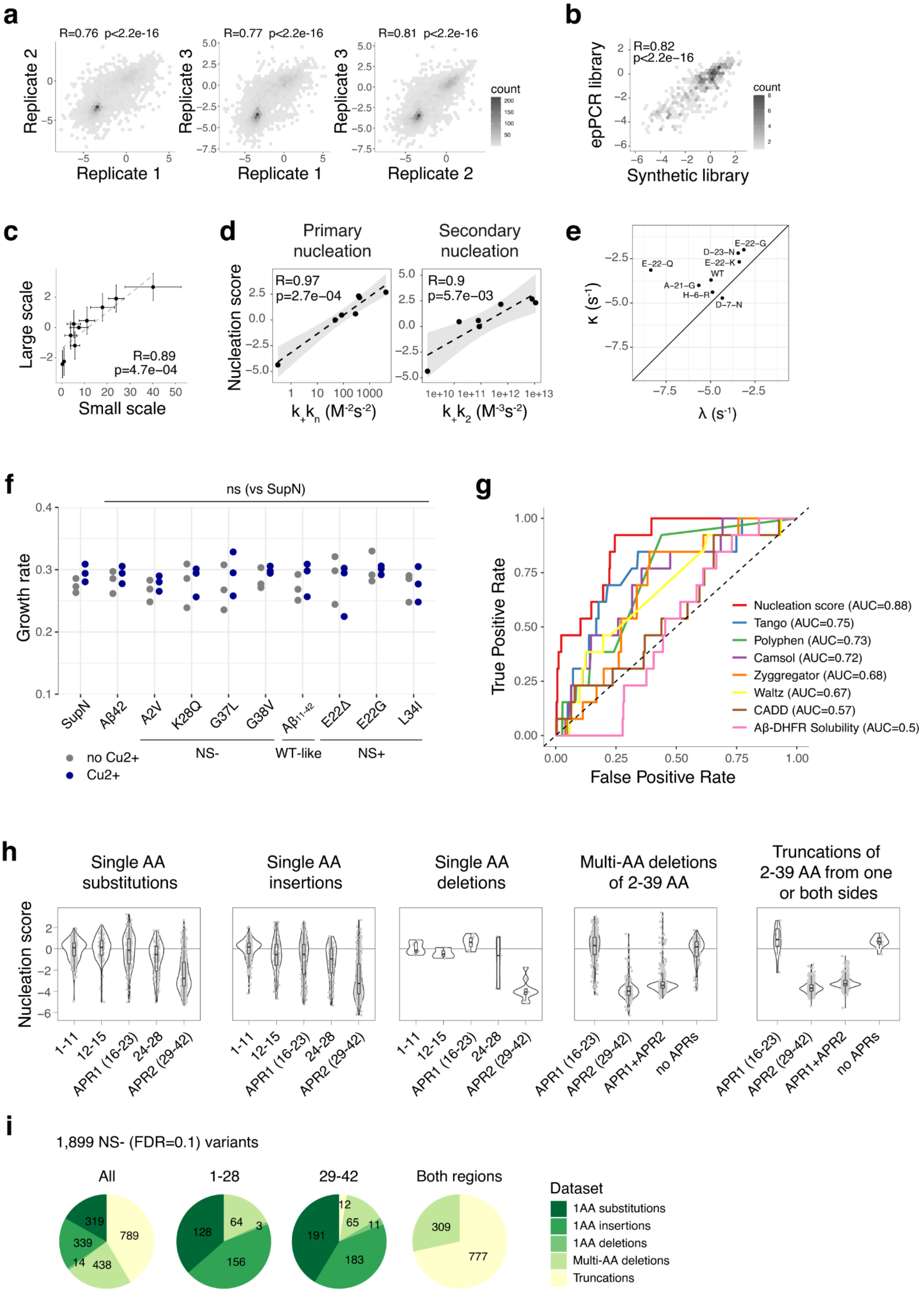
a
R=0.76 p<2.2e-16
Replicate 2
Replicate 1
R=0.77 p<2.2e-16
Replicate 3
Replicate 1
R=0.81 p<2.2e-16
Replicate 3
Replicate 2
count
b
R=0.82
p<2.2e−16
epPCR library
Synthetic library
count
c
Large scale
Small scale
R=0.89
p=4.7e−04
d
Primary nucleation
Secondary nucleation
Nucleation score
R=0.97
p=2.7e−04
R=0.9
p=5.7e−03
k_+k_n ($M^{-2}s^{-2}$)
k_+k_2 ($M^{-3}s^{-2}$)
e
E−22−G
D−23−N
E−22−Q
E−22−K
WT
A−21−G
H−6−R
D−7−N
κ (s^{-1})
λ (s^{-1})
f
ns (vs SupN)
Growth rate
SupN
Aβ42
A2V
K28Q
G37L
G38V
$Aβ_{11-42}$
E22Δ
E22G
L34I
NS-
WT-like
NS+
no Cu2+
Cu2+
g
True Positive Rate
False Positive Rate
Nucleation score (AUC=0.88)
Tango (AUC=0.75)
Polyphen (AUC=0.73)
Camsol (AUC=0.72)
Zyggregator (AUC=0.68)
Waltz (AUC=0.67)
CADD (AUC=0.57)
Aβ-DHFR Solubility (AUC=0.5)
h
Single AA substitutions
Single AA insertions
Single AA deletions
Multi-AA deletions of 2-39 AA
Truncations of 2-39 AA from one or both sides
Nucleation score
1-11
12-15
APR1 (16-23)
24-28
APR2 (29-42)
APR1+APR2
no APRs
i
1,899 NS- (FDR=0.1) variants
All
1-28
29-42
Both regions
319
339
14
438
789
128
64
3
156
12
191
65
11
183
309
777
Dataset
1AA substitutions
1AA insertions
1AA deletions
Multi-AA deletions
Truncations

Supplementary Fig. 1. Reproducibility and assay validation

a Correlation of nucleation scores for three biological replicates ($n_{1\text{-}2}$=2,951, $n_{1\text{-}3}$=2,984, $n_{2\text{-}3}$=2,950 genotypes) **b** Correlation of nucleation scores measured for the synthetic library used in this study and a previous library generated by error-prone PCR (n=423 common variants)[1] (p=2.6e-106). **c** Correlation of nucleation scores measured in the competition experiment or individually for selected variants (n=10). Vertical and horizontal error bars indicate 95% confidence intervals of mean NS. Pearson correlation coefficients are indicated in (**a-c**). **d** Correlation of nucleation scores with *in vitro* primary and secondary nucleation combined rate constants from Tht *in vitro* measurements[2]. Weighted Pearson correlation coefficients are indicated. Gray band indicates 95% confidence interval of the linear fits. **e** κ vs λ rate constants from HDX-MS measurements[3] **f** Individually measured growth rates for selected variants increasing (NS+), decreasing (NS-) or having no effect (WT-like) at FDR=0.1, in non-inducing (no Cu2+) and inducing (Cu2+) protein expression conditions (n=3 biological replicates/variant). One-way ANOVA with Dunnett's multiple comparisons test against SupN Cu2+. **g** Receiver operating characteristic (ROC) curves for 13 of all the single AA substitutions described as dominant fAD variants (H6R, D7N, D7H, E11K, K16Q, L17V, A21G, E22Q, E22K, E22G, D23N, L34V and A42T) versus all other single AA substitutions present in the dataset ($n_{non\text{-}fAD}$=738) for two DMS datasets (Nucleation score, estimated by fusing SupN-Aß, and Solubility scores[4], estimated by fusing Aß variants to an enzyme required for growth, DHFR), aggregation predictors (Tango, Zyggregator, Waltz, Camsol[5-8]) and variant effect predictors (Polyphen and CADD10[9,10]). Area under the curve (AUC) values are indicated. Diagonal dashed line indicates the performance of a random classifier. **h** Distributions of nucleation scores for mutations in different regions as reported in[5,11] ($n_{1\text{-}11\ subs}$=201, $n_{12\text{-}15\ subs}$=69, $n_{16\text{-}23\ subs}$=141, $n_{24\text{-}28\ subs}$=90, $n_{29\text{-}42\ subs}$= 250, $n_{1\text{-}11\ ins}$=205, $n_{12\text{-}15\ ins}$=73, $n_{16\text{-}23\ ins}$=150, $n_{24\text{-}28\ ins}$=94, $n_{29\text{-}42\ ins}$=241, $n_{1\text{-}11\ s.del}$=11, $n_{12\text{-}15\ s.del}$=3, $n_{16\text{-}23\ s.del}$=7, $n_{24\text{-}28\ s.del}$=5, $n_{29\text{-}42\ s.del}$=11, $n_{16\text{-}23\ multi\text{-}del}$=237, $n_{29\text{-}42\ multi\text{-}del}$=125, $n_{APR1+APR2\ multi\text{-}del}$=271, $n_{no\ APR\ multi\text{-}del}$=96, $n_{16\text{-}23\ trunc}$=13, $n_{29\text{-}42\ truncl}$=303, $n_{APR1+APR2\ trunc}$=487, $n_{no\ APR\ trunc}$=14 genotypes). Boxplots represent median values and the lower and upper hinges correspond to the 25th and 75th percentiles, respectively. Whiskers extend from the hinge to the largest value no further than 1.5*interquartile range. **i** Number and type of variants increasing nucleation (NS-, FDR=0.1) for each peptide region. Source data are provided as a Source Data file.

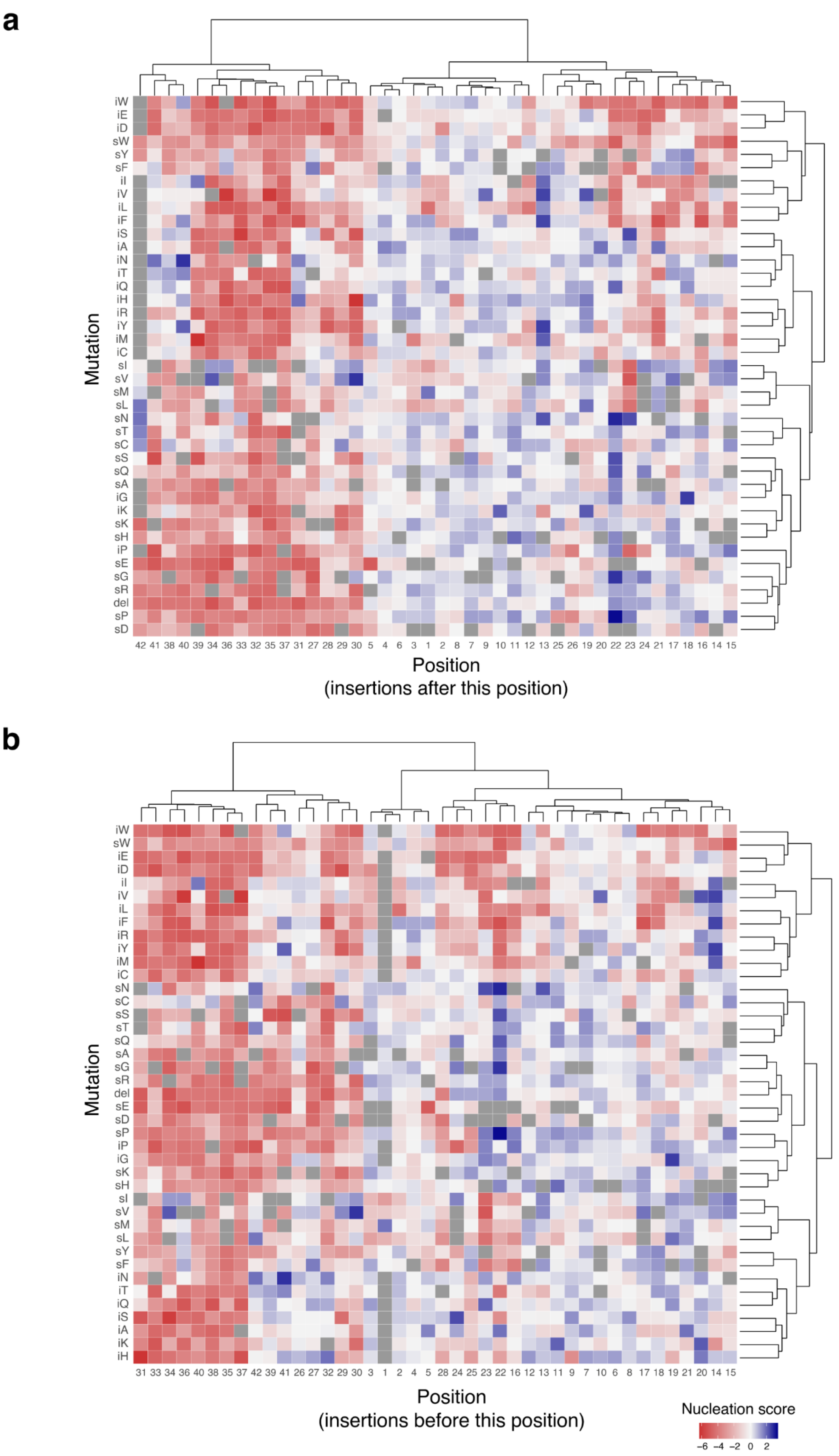
a
Mutation
iW iE iD sW sY sF iI iV iL iF iS iA iN iT iQ iH iR iY iM iC sI sV sM sL sN sT sC sS sQ sA iG iK sK sH iP sE sG sR del sP sD
42 41 38 40 39 34 36 33 32 35 37 31 27 28 29 30 5 4 6 3 1 2 8 7 9 10 11 12 13 25 26 19 20 22 23 24 21 17 18 16 14 15
Position
(insertions after this position)
b
Mutation
iW sW iE iD iI iV iL iF iR iY iM iC sN sC sS sT sQ sA sG sR del sE sD sP iP iG sK sH sI sV sM sL sY sF iN iT iQ iS iA iK iH
31 33 34 36 40 38 35 37 42 39 41 26 27 32 29 30 3 1 2 4 5 28 24 25 23 22 16 12 13 11 9 7 10 6 8 17 18 19 21 20 14 15
Position
(insertions before this position)
Nucleation score
−6 −4 −2 0 2

Supplementary Fig. 2. Mutational effects of single AA substitutions and insertions

a,b Clustering of single AA mutation nucleation scores by mutated residue identity and position. Position is indicated in the x-axis; AA insertions were considered after (**a**) or before (**b**) each position. Mutations are indicated in the y-axis and labeled with an ‘s’ for substitutions or an ‘i’ for insertions, followed by the substituted or inserted AA. ‘del’ indicates single AA deletion of that position.

a

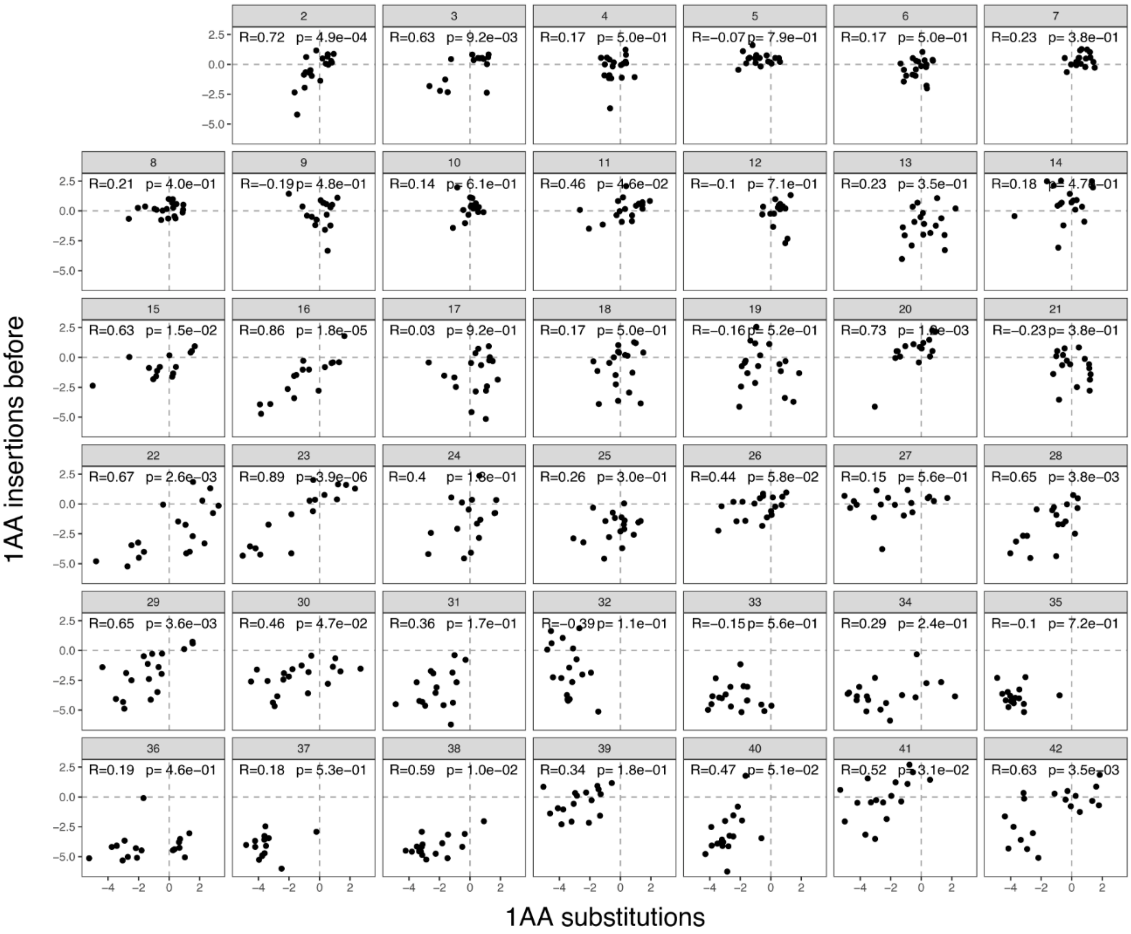
1AA insertions before
1AA substitutions

b

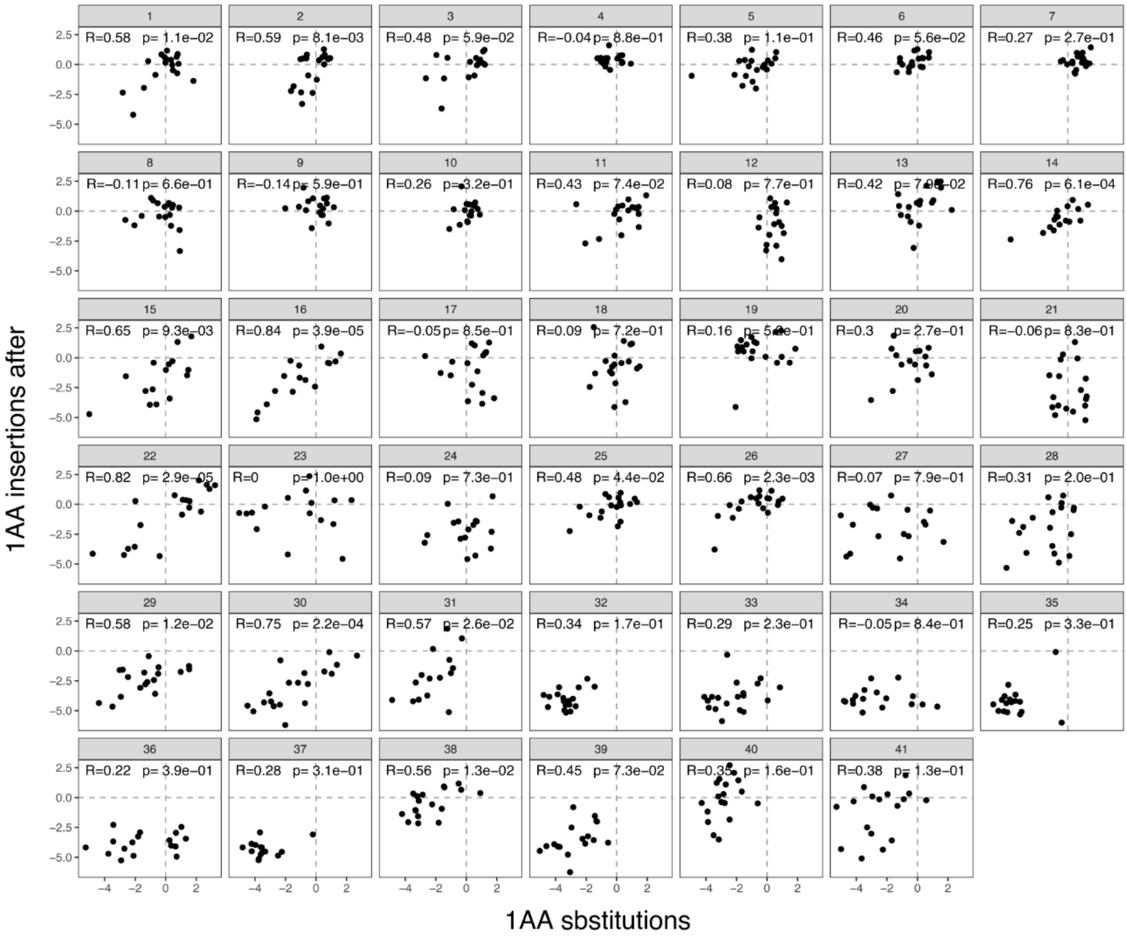
1AA insertions after
1AA sbstitutions

Supplementary Fig. 3. Comparing the mutational effects of single AA substitutions and insertions

a,b Correlation of nucleation scores at each position arranged by each AA type, between single AA substitutions and single AA insertions before (**a**) or after (**b**) the corresponding position. Pearson correlation coefficients are indicated. Dashed lines indicate the WT nucleation score (0).

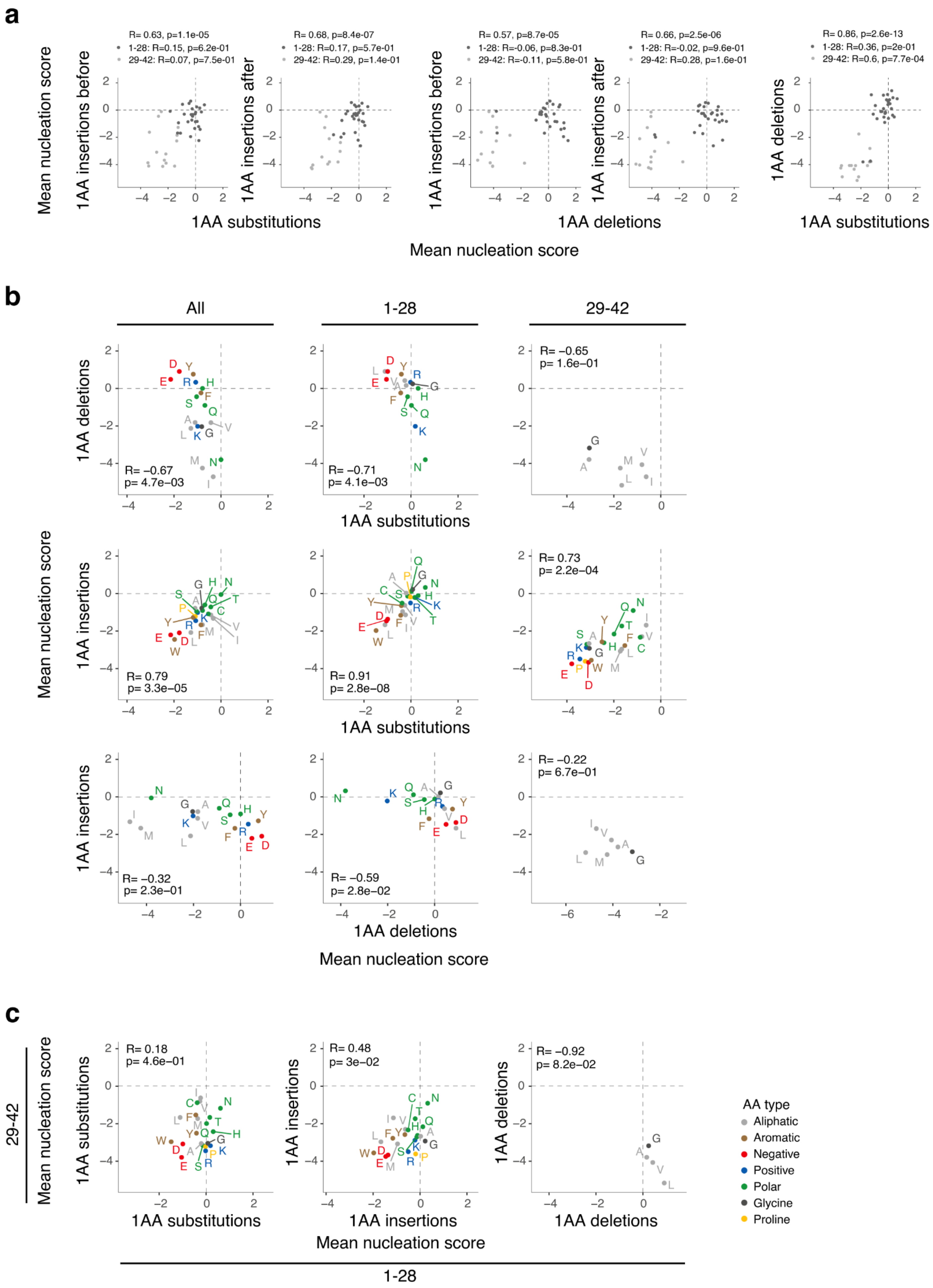
a
R= 0.63, p=1.1e-05
1-28: R=0.15, p=6.2e-01
29-42: R=0.07, p=7.5e-01
R= 0.68, p=8.4e-07
1-28: R=0.17, p=5.7e-01
29-42: R=0.29, p=1.4e-01
R= 0.57, p=8.7e-05
1-28: R=-0.06, p=8.3e-01
29-42: R=-0.11, p=5.8e-01
R= 0.66, p=2.5e-06
1-28: R=-0.02, p=9.6e-01
29-42: R=0.28, p=1.6e-01
R= 0.86, p=2.6e-13
1-28: R=0.36, p=2e-01
29-42: R=0.6, p=7.7e-04
Mean nucleation score
1AA insertions before
1AA insertions after
1AA insertions before
1AA insertions after
1AA deletions
1AA substitutions
1AA deletions
1AA substitutions
Mean nucleation score
b
All
1-28
29-42
1AA deletions
R= −0.67
p= 4.7e−03
R= −0.71
p= 4.1e−03
R= −0.65
p= 1.6e−01
1AA substitutions
Mean nucleation score
1AA insertions
R= 0.79
p= 3.3e−05
R= 0.91
p= 2.8e−08
R= 0.73
p= 2.2e−04
1AA substitutions
1AA insertions
R= −0.32
p= 2.3e−01
R= −0.59
p= 2.8e−02
R= −0.22
p= 6.7e−01
1AA deletions
Mean nucleation score
c
29-42
Mean nucleation score
1AA substitutions
R= 0.18
p= 4.6e−01
1AA insertions
R= 0.48
p= 3e−02
1AA deletions
R= −0.92
p= 8.2e−02
1AA substitutions
1AA insertions
1AA deletions
Mean nucleation score
1-28
AA type
Aliphatic
Aromatic
Negative
Positive
Polar
Glycine
Proline

Supplementary Fig. 4. Comparing the mutational effects of single AA variants

a Correlation of average nucleation scores for each position, for single AA insertions before or after a specific position and single AA substitutions (left) or single AA deletions (middle), and for single AA deletions and single AA substitutions (right) at the corresponding position. Color code indicates peptide region (AA 1-28 or AA 29-42). **b** Correlation of average nucleation scores for each AA, for single AA deletions and single AA substitutions (top row), single AA insertions and single AA substitutions (middle row) and single AA insertions and single AA deletions (bottom row); and for the full peptide (left column), the N-terminal region (AA 1-28, middle column) or the C-terminal region (AA 29-42, right column). **c** Correlation of average nucleation scores for each AA, for the C and the N-terminal regions, for single AA substitutions (left), single AA insertions (middle) and single AA deletions (right). AA labels are coloured by AA class in (**b**) and (**c**). Pearson correlation coefficients are indicated. Dashed lines indicate the WT nucleation score (0).

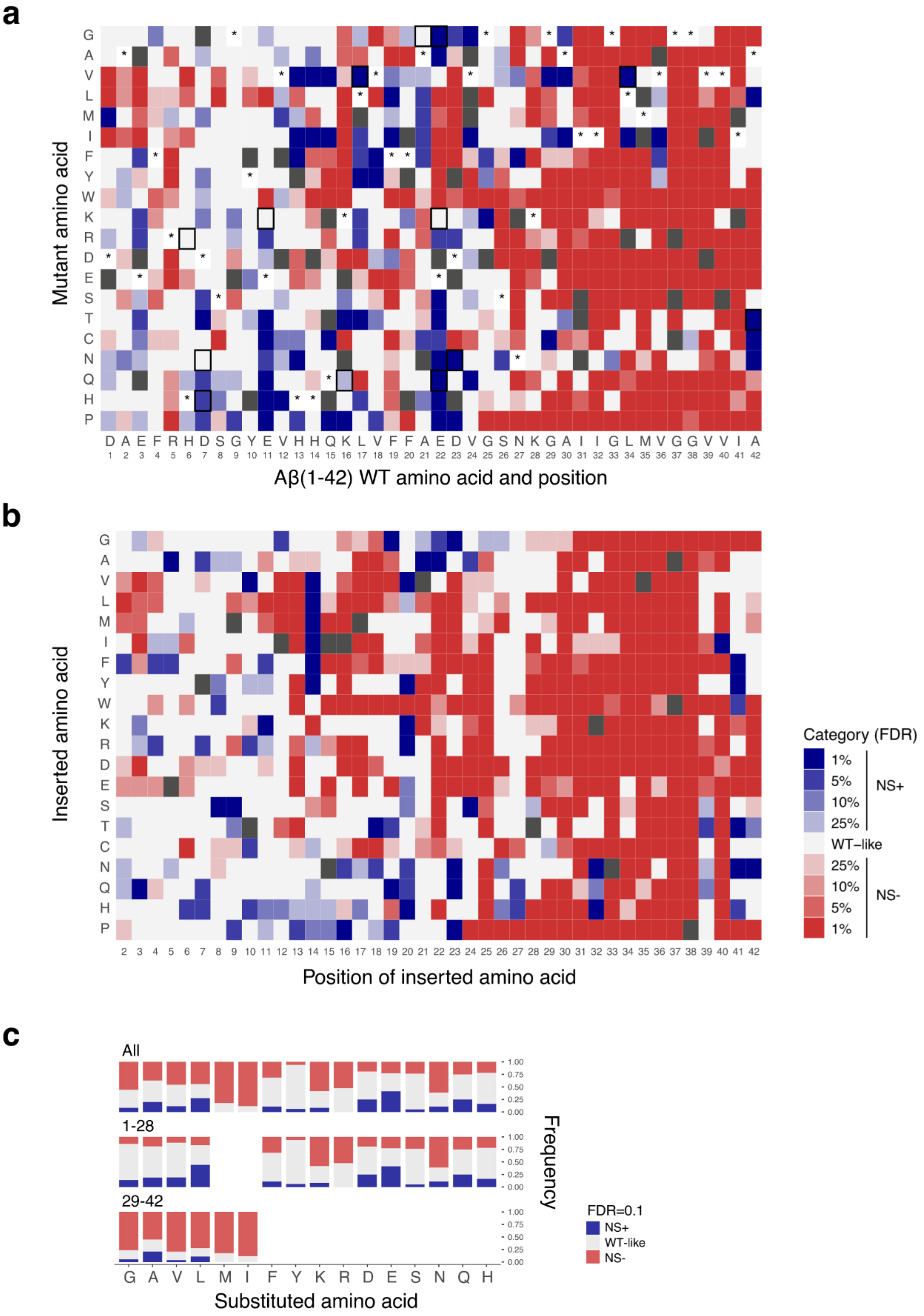

Supplementary Fig. 5. Mutational effects of single AA substitutions and insertions

a Heatmap of nucleation scores FDR categories for single AA substitutions. The WT AA and position are indicated in the x-axis and the mutant AA is indicated in the y-axis. Variants not present are represented in gray. Synonymous mutants are indicated with '*' and fAD mutants with a black box. **b** Heatmap of nucleation scores FDR categories for single AA insertions. **c** Frequency of increasing or decreasing nucleation (FDR=0.1) single AA substitutions upon substituting specific WT AA, for each peptide region.

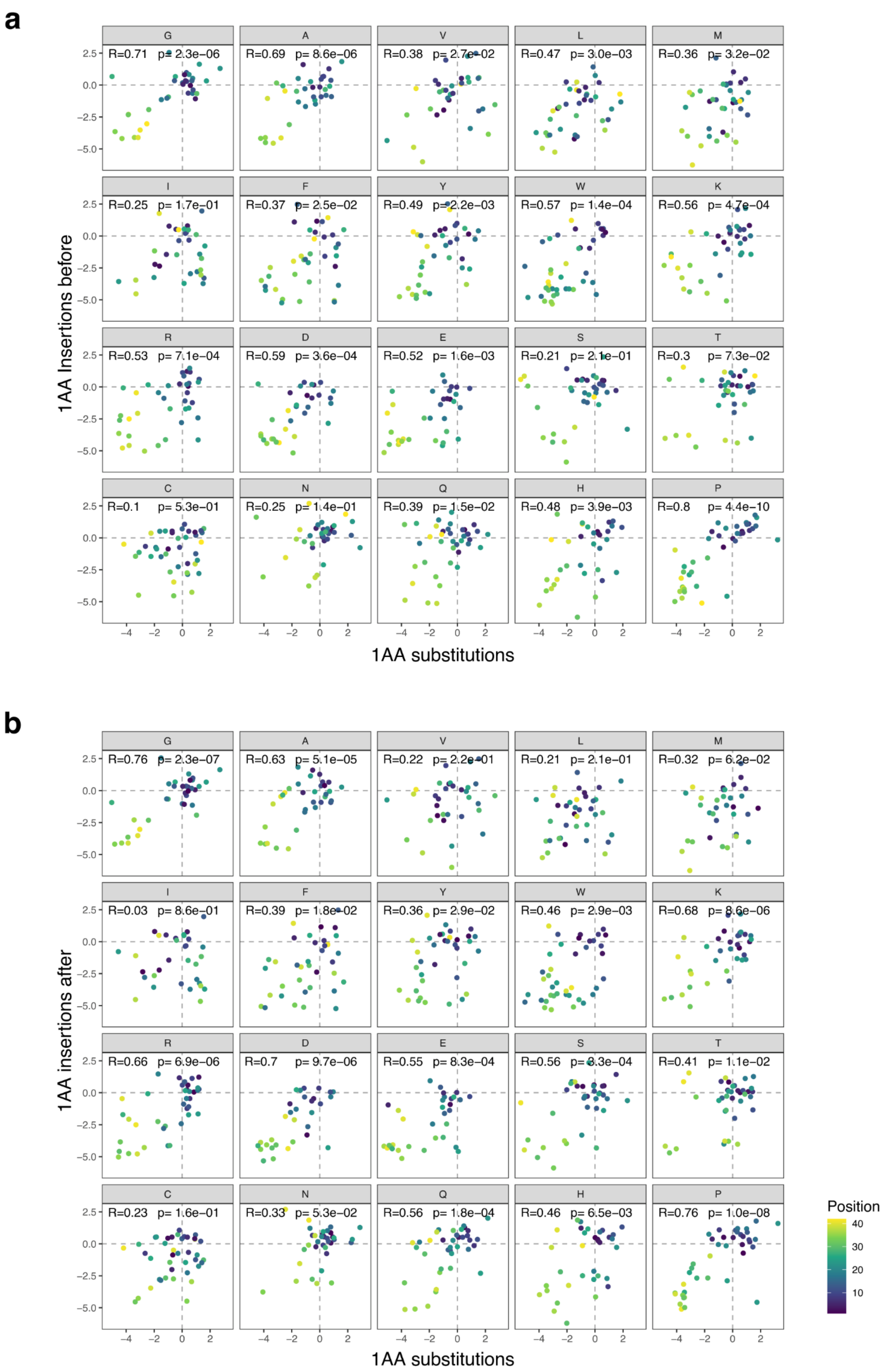
a
G R=0.71 p= 2.3e−06
A R=0.69 p= 8.6e−06
V R=0.38 p= 2.7e−02
L R=0.47 p= 3.0e−03
M R=0.36 p= 3.2e−02
I R=0.25 p= 1.7e−01
F R=0.37 p= 2.5e−02
Y R=0.49 p= 2.2e−03
W R=0.57 p= 1.4e−04
K R=0.56 p= 4.7e−04
R R=0.53 p= 7.1e−04
D R=0.59 p= 3.6e−04
E R=0.52 p= 1.6e−03
S R=0.21 p= 2.1e−01
T R=0.3 p= 7.3e−02
C R=0.1 p= 5.3e−01
N R=0.25 p= 1.4e−01
Q R=0.39 p= 1.5e−02
H R=0.48 p= 3.9e−03
P R=0.8 p= 4.4e−10
1AA Insertions before
1AA substitutions
b
G R=0.76 p= 2.3e−07
A R=0.63 p= 5.1e−05
V R=0.22 p= 2.2e−01
L R=0.21 p= 2.1e−01
M R=0.32 p= 6.2e−02
I R=0.03 p= 8.6e−01
F R=0.39 p= 1.8e−02
Y R=0.36 p= 2.9e−02
W R=0.46 p= 2.9e−03
K R=0.68 p= 8.6e−06
R R=0.66 p= 6.9e−06
D R=0.7 p= 9.7e−06
E R=0.55 p= 8.3e−04
S R=0.56 p= 8.3e−04
T R=0.41 p= 1.1e−02
C R=0.23 p= 1.6e−01
N R=0.33 p= 5.3e−02
Q R=0.56 p= 1.8e−04
H R=0.46 p= 6.5e−03
P R=0.76 p= 1.0e−08
1AA insertions after
1AA substitutions
Position
40
30
20
10

Supplementary Fig. 6. Comparing the mutational effects of single AA substitutions and insertions

a,b Correlation of nucleation scores for each AA type arranged by position, between single AA substitutions and single AA insertions before (**a**) or after (**b**) the corresponding position. Pearson correlation coefficients are indicated. Dashed lines indicate the WT nucleation score (0). Color code indicates AA position.

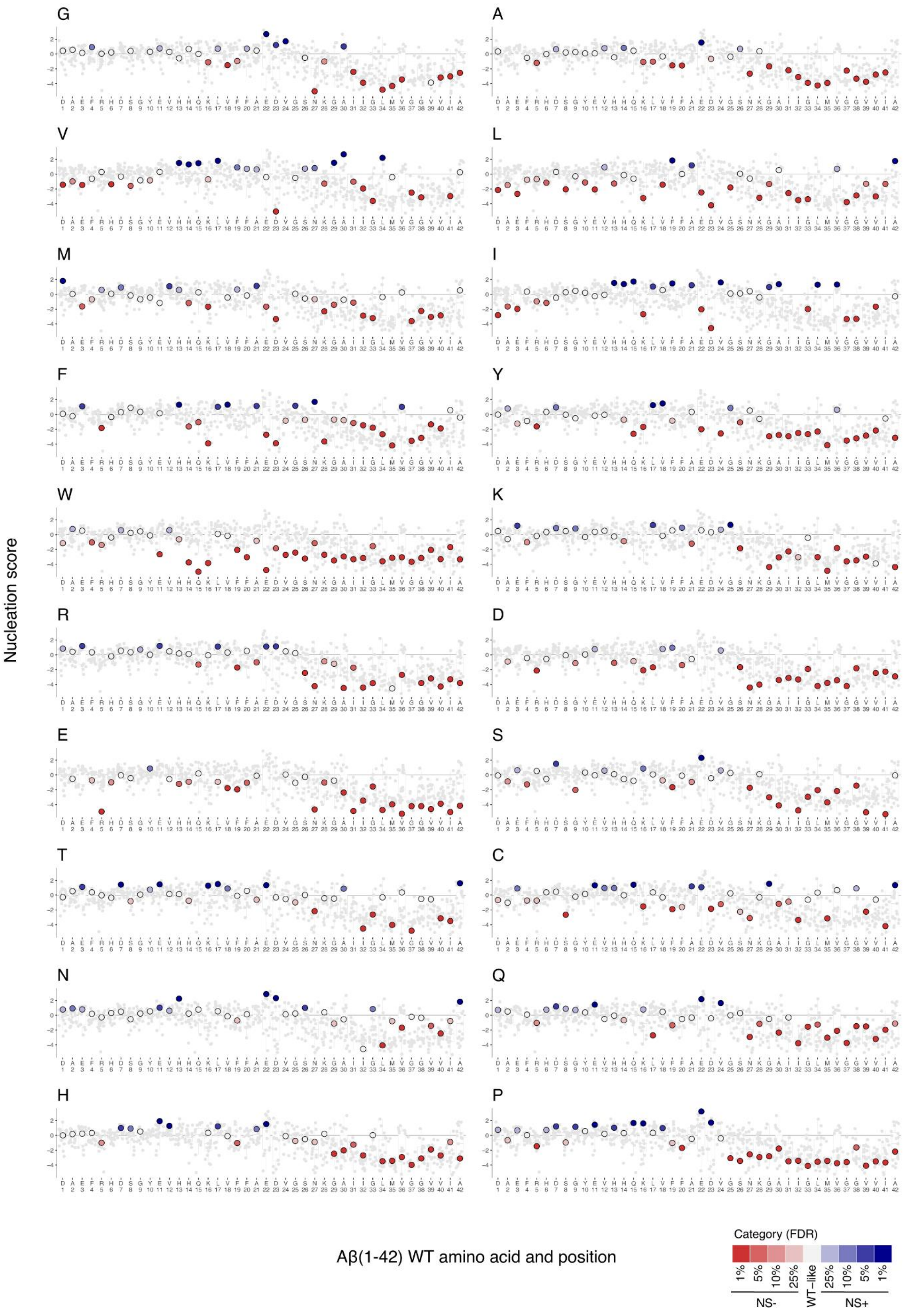

Supplementary Fig. 7. Mutational effects of substituting in specific AAs

The wild-type (WT) AA and position are indicated on the x-axis. Color code indicates FDR category. The horizontal line indicates the WT nucleation score (0).

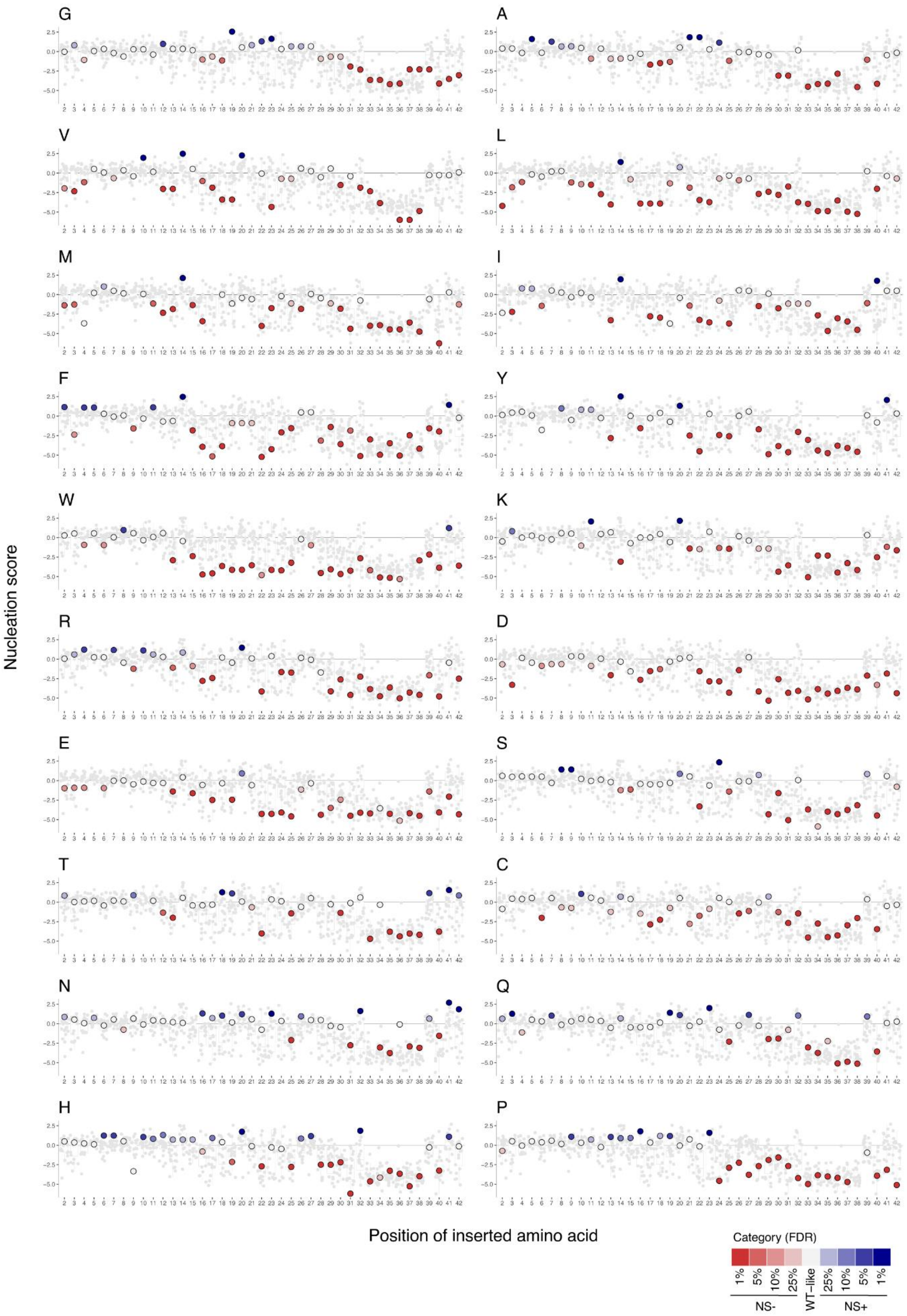

Supplementary Fig. 8. Mutational effects of inserting specific AAs

The wild-type (WT) AA and position are indicated on the x-axis. Color code indicates FDR category. The horizontal line indicates the WT nucleation score (0).

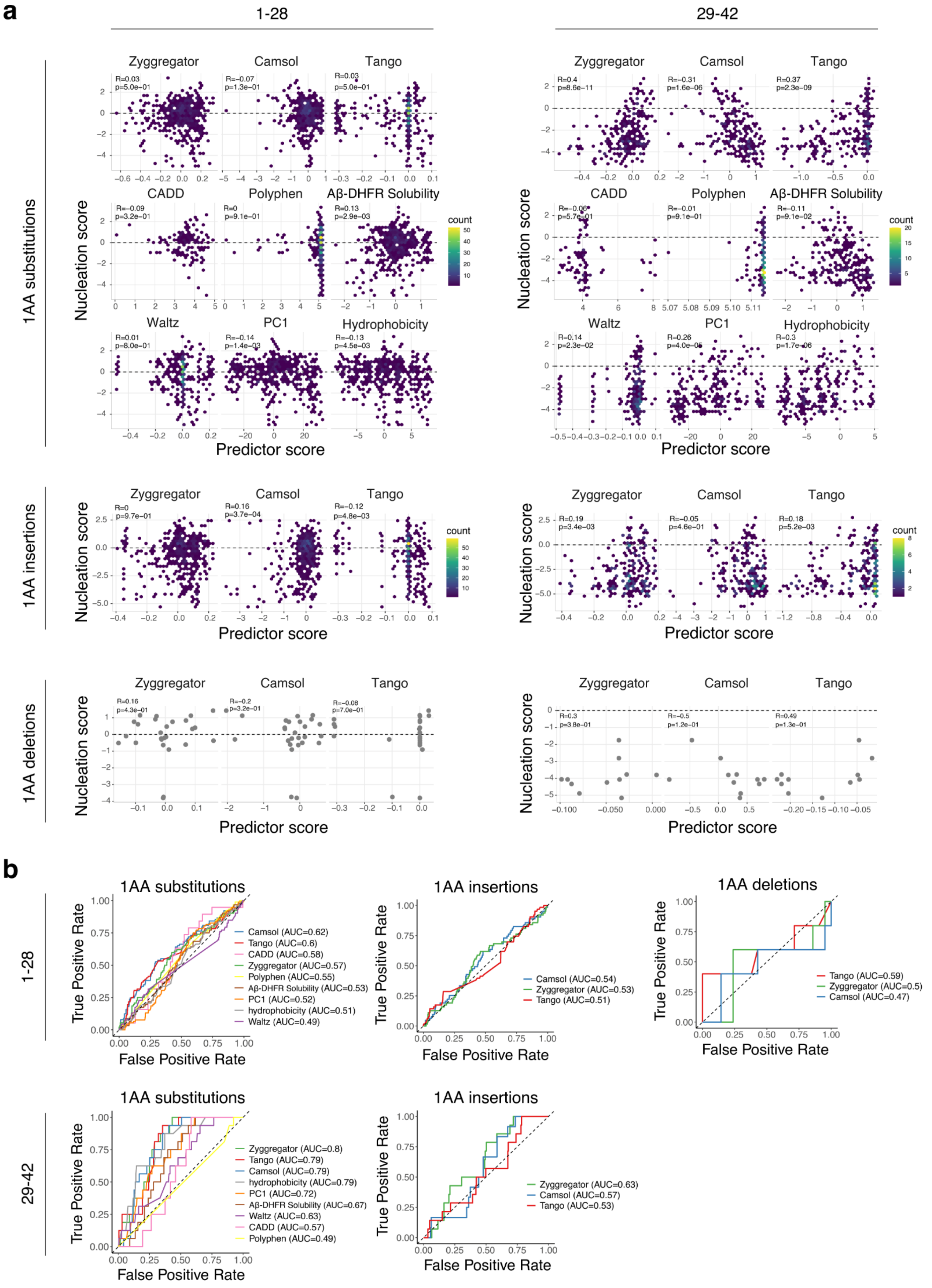

a
1-28
29-42
1AA substitutions
Zyggregator
Camsol
Tango
CADD
Polyphen
Aβ-DHFR Solubility
Waltz
PC1
Hydrophobicity
Nucleation score
Predictor score
count
1AA insertions
1AA deletions
b
1AA substitutions
1AA insertions
1AA deletions
True Positive Rate
False Positive Rate
Camsol (AUC=0.62)
Tango (AUC=0.6)
CADD (AUC=0.58)
Zyggregator (AUC=0.57)
Polyphen (AUC=0.55)
Aβ-DHFR Solubility (AUC=0.53)
PC1 (AUC=0.52)
hydrophobicity (AUC=0.51)
Waltz (AUC=0.49)
Camsol (AUC=0.54)
Zyggregator (AUC=0.53)
Tango (AUC=0.51)
Tango (AUC=0.59)
Zyggregator (AUC=0.5)
Camsol (AUC=0.47)
Zyggregator (AUC=0.8)
Tango (AUC=0.79)
Camsol (AUC=0.79)
hydrophobicity (AUC=0.79)
PC1 (AUC=0.72)
Aβ-DHFR Solubility (AUC=0.67)
Waltz (AUC=0.63)
CADD (AUC=0.57)
Polyphen (AUC=0.49)
Zyggregator (AUC=0.63)
Camsol (AUC=0.57)
Tango (AUC=0.53)

Supplementary Fig. 9. Evaluation of mutational effect and aggregation predictors

a Correlation of nucleation scores with the predictions of aggregation predictors (Tango, Zyggregator, Waltz and Camsol)[5-8] variant effect predictors (CADD, Polyphen)[9,10], solubility scores[4], PC1[12] and hydrophobicity[13] for single AA mutations, at the N-terminus (AA 1-28, left) or the C-terminus (AA 29-42, right). Pearson correlation coefficients are indicated. Dashed lines indicate the WT nucleation score (0). **b** Receiver operating characteristic (ROC) curves for classifying increasing nucleation variants (NS+, FDR=0.1) for single AA mutations, at the N and C-terminal regions, for aggregation predictors[5-8], variant effect predictors[9,10], solubility scores estimated by fusing Aß variants to an enzyme required for growth, DHFR[4], PC1[12] and hydrophobicity[13]. Area under the curve (AUC) values are indicated. Diagonal dashed line indicates the performance of a random classifier.

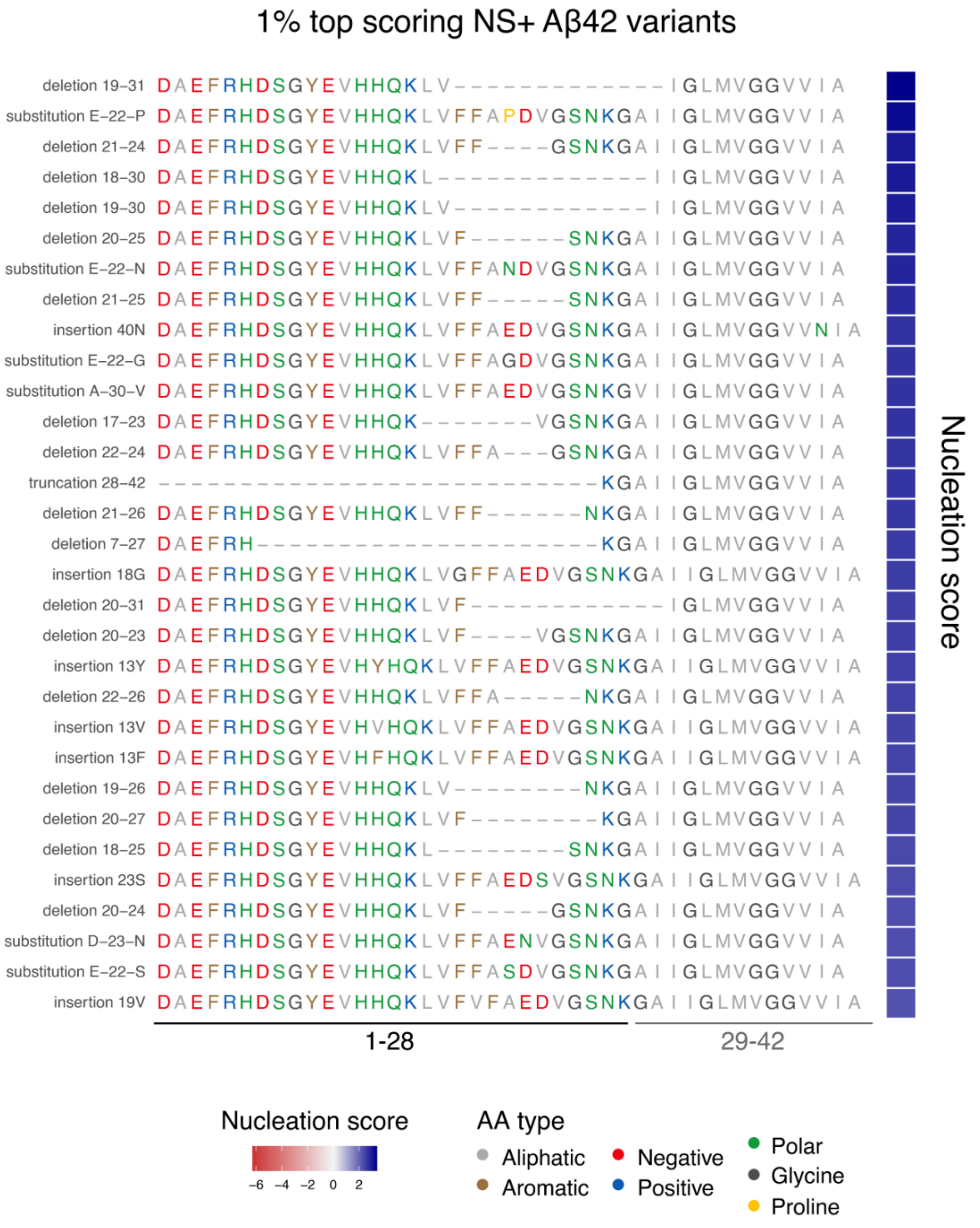

Supplementary Fig. 10. Top nucleating sequences in the library

AA sequence for 1% variants with highest NS (all FDR=0.1) in the library. AA are coloured by AA class.

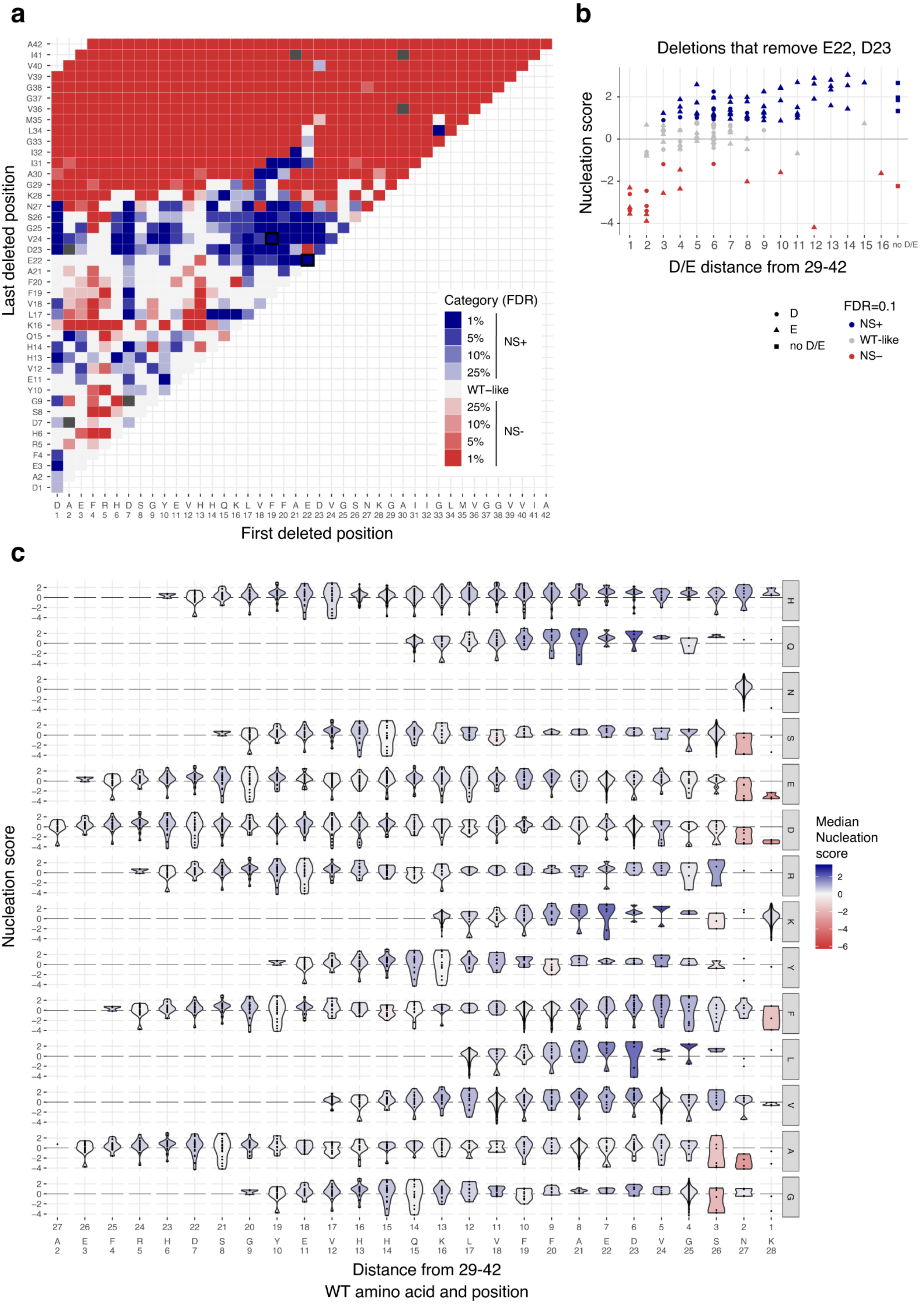
a
Last deleted position
First deleted position
Category (FDR)
1%
5%
10%
25%
NS+
WT−like
25%
10%
5%
1%
NS-
b
Deletions that remove E22, D23
Nucleation score
D/E distance from 29-42
no D/E
D
E
no D/E
FDR=0.1
NS+
WT-like
NS−
c
Nucleation score
Median Nucleation score
Distance from 29-42
WT amino acid and position

Supplementary Fig. 11. Multi-AA deletions

a Heatmap of nucleation scores FDR categories for multi-AA deletions. The WT AA and position of the first and last residues deleted are indicated in the x-axis and y-axis, respectively. The black squares indicate fAD variants: Osaka (E22Δ) and Uppsala (Δ19-24). Variants not present are represented in gray. **b** Effect on nucleation of variants that delete E22, D23N or both. The distance the closest negative residue (D,E) - if present - to the C-terminus (AA 29-42) is shown in the x-axis. Variants with no negative residues are also shown (no D/E). Shape indicates the identity of the residue and color code indicates FDR=0.1 category. The horizontal line indicates the WT nucleation score (0). **c** Generation of new N-terminus sequences flanking the Aß core. Nucleation score distributions of each AA at each position for deletions at the N-terminus (AA 1-28). Distance from the C-terminus (AA 29-42) is indicated in the x-axis, as well as WT AA and position. Color of the violin plot indicates median nucleation score for each distribution.

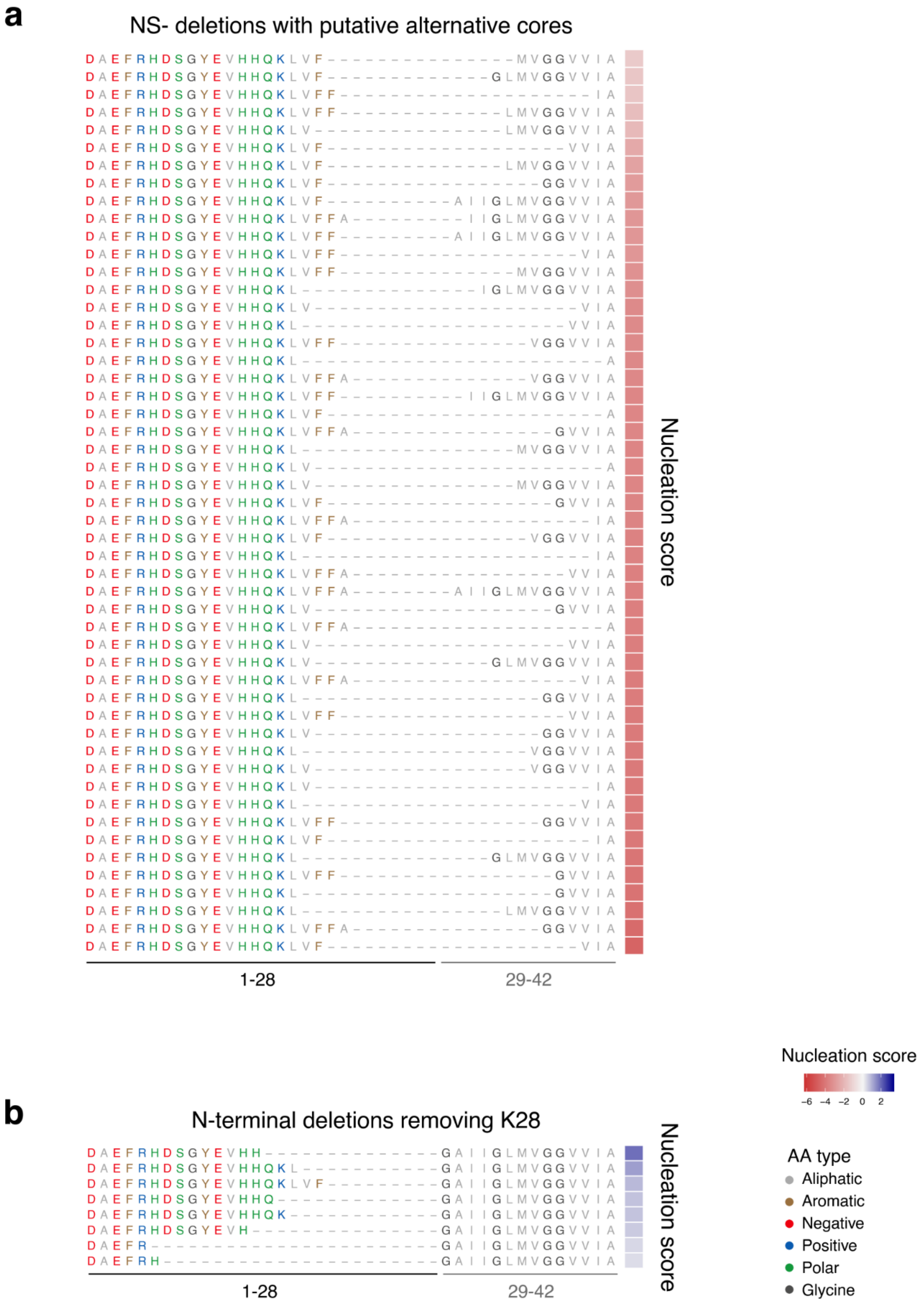

Supplementary Fig. 12. Multi-AA deletion variants

a AA sequence for variants with internal multi-AA deletions located at both N and C-terminus, with significantly decreased nucleation (FDR=0.1). **b** AA sequence for deletions in the N-terminus that remove residue K28, with positive nucleation score (NS>0).

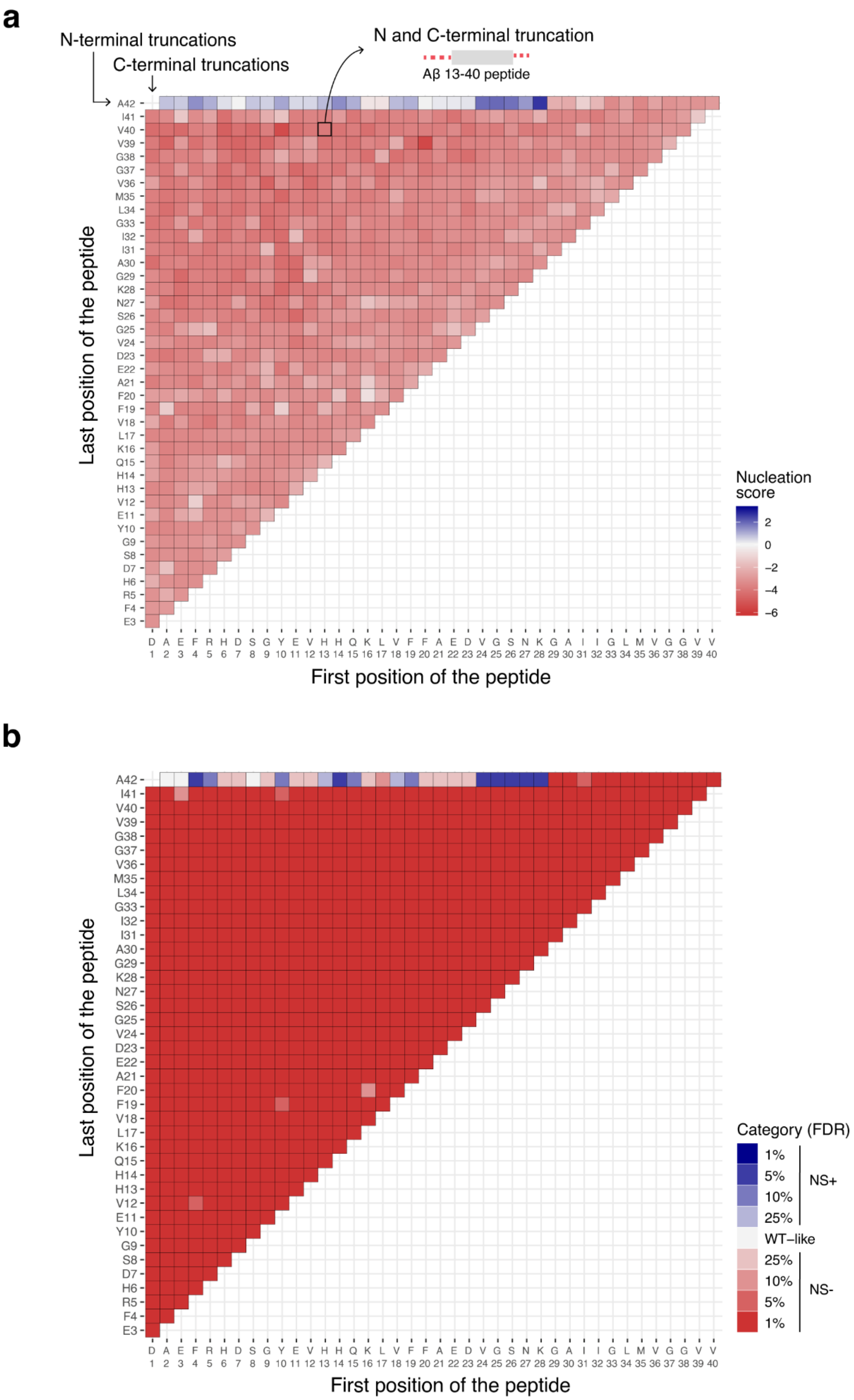

Supplementary Fig. 13. N- and C-terminal truncations

a,b Heatmap of nucleation scores (**a**) or FDR categories (**b**) for truncations from one or both ends of the peptide. The WT AA and position of the first and last residues of the resulting peptide are indicated in the x-axis and y-axis, respectively.

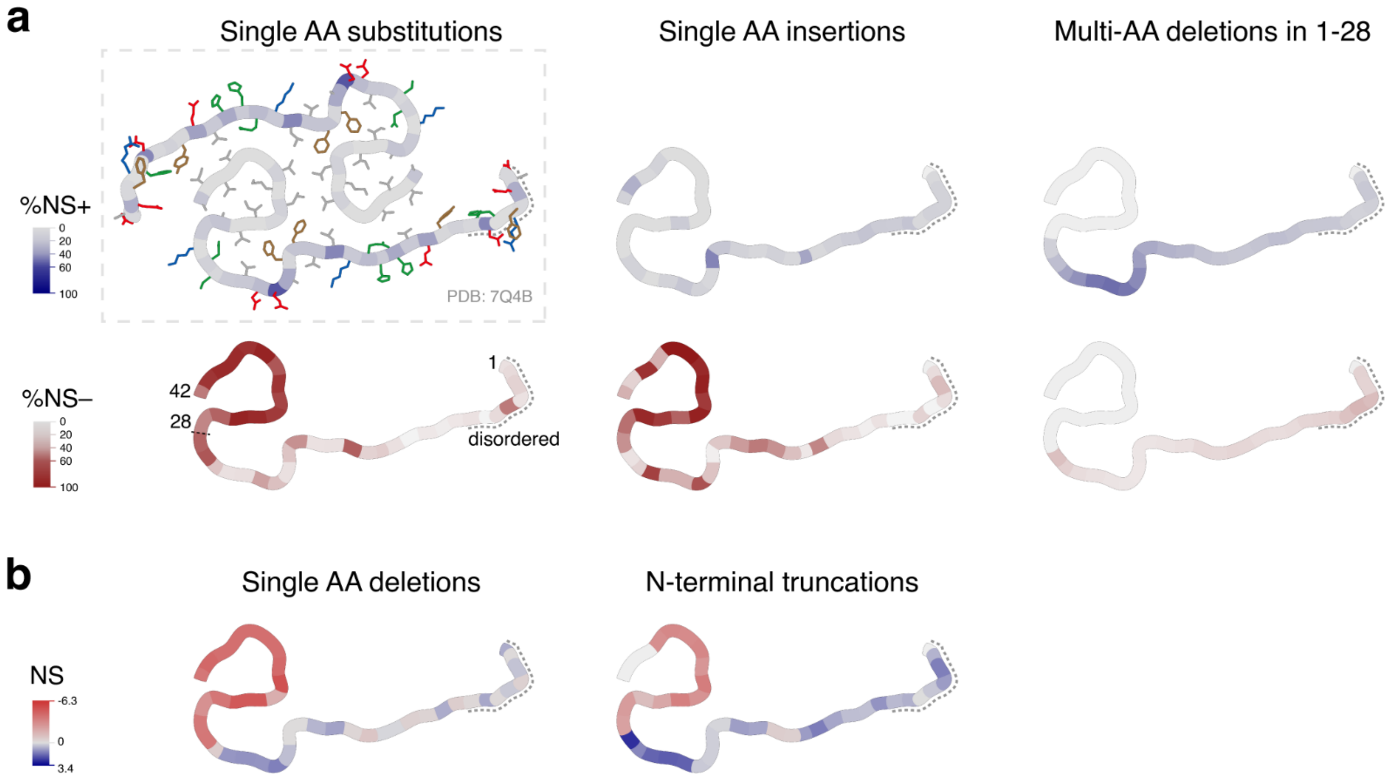

Supplementary Fig. 14. Impact of diverse classes of mutations along the structure of Aß42 fibrils from sporadic AD brains

The impact of all mutations of all classes is summarized over the structure of Aß42 fibrils (PDB: 7Q4B [https://doi.org/10.2210/pdb7Q4B/pdb])[14]. In fibrils extracted from sporadic AD brains, Aß42 adopts a S-shaped structure at the C-terminus with an N-terminal arm linking to an unstructured region (AA 1-9). **a** Single AA substitutions, single AA insertions and multi-AA deletions in AA 1-28: Color intensity indicates the percentage of NS+ (blue) or NS- (red) mutations at each position or losing each position (for multi-AA deletions) (FDR=0.1). Side chain colored by AA class (red: negative, blue: positive, green: polar, gray: aliphatic, brown: aromatic). **b** Single AA deletions and N-terminal truncations: Color intensity depicts the nucleation score of each single AA deletion or of the N-terminal truncation starting at that position. White depicts positions that are not mutated in each dataset.

Supplementary References

1. Seuma, M., Faure, A., Badia, M., Lehner, B. & Bolognesi, B. The genetic landscape for amyloid beta fibril nucleation accurately discriminates familial Alzheimer's disease mutations. *Elife* **10**, e63364 (2021).

2. Thacker, D. *et al.* The role of fibril structure and surface hydrophobicity in secondary nucleation of amyloid fibrils. *Proc. Natl. Acad. Sci. U. S. A.* **117**, 25272–25283 (2020).

3. Illes-Toth, E., Meisl, G., Rempel, D. L., Knowles, T. P. J. & Gross, M. L. Pulsed Hydrogen-Deuterium Exchange Reveals Altered Structures and Mechanisms in the Aggregation of Familial Alzheimer's Disease Mutants. *ACS Chem. Neurosci.* **12**, 1972–1982 (2021).

4. Gray, V. E. *et al.* Elucidating the Molecular Determinants of Aβ Aggregation with Deep Mutational Scanning. *G3* **9**, 3683–3689 (2019).

5. Fernandez-Escamilla, A.-M., Rousseau, F., Schymkowitz, J. & Serrano, L. Prediction of sequence-dependent and mutational effects on the aggregation of peptides and proteins. *Nat. Biotechnol.* **22**, 1302–1306 (2004).

6. Maurer-Stroh, S. *et al.* Exploring the sequence determinants of amyloid structure using position-specific scoring matrices. *Nat. Methods* **7**, 237–242 (2010).

7. Tartaglia, G. G. & Vendruscolo, M. The Zyggregator method for predicting protein aggregation propensities. *Chem. Soc. Rev.* **37**, 1395–1401 (2008).

8. Sormanni, P., Aprile, F. A. & Vendruscolo, M. The CamSol method of rational design of protein mutants with enhanced solubility. *J. Mol. Biol.* **427**, 478–490 (2015).

9. Rentzsch, P., Witten, D., Cooper, G. M., Shendure, J. & Kircher, M. CADD: predicting the deleteriousness of variants throughout the human genome. *Nucleic Acids Res.* **47**, D886–D894 (2019).

10. Adzhubei, I., Jordan, D. M. & Sunyaev, S. R. Predicting functional effect of human missense mutations using PolyPhen-2. *Curr. Protoc. Hum. Genet.* **Chapter 7**, Unit7.20 (2013).

11. van der Kant, R., Louros, N., Schymkowitz, J. & Rousseau, F. Thermodynamic analysis of amyloid fibril structures reveals a common framework for stability in amyloid polymorphs. *Structure* (2022) doi:10.1016/j.str.2022.05.002.

12. Bolognesi, B. *et al.* The mutational landscape of a prion-like domain. *Nat. Commun.* **10**, 4162 (2019).

13. Kyte, J. & Doolittle, R. F. A simple method for displaying the hydropathic character of a protein. *J. Mol. Biol.* **157**, 105–132 (1982).

14. Yang, Y. *et al.* Cryo-EM structures of amyloid-β 42 filaments from human brains. *Science* **375**, 167–172 (2022).
