# Supplementary material for: An atlas of amyloid aggregation: the impact of substitutions, insertions, deletions and truncations on amyloid beta fibril nucleation
Source: Nat Commun. 2022 Nov 18;13:7084. doi: 10.1038/s41467-022-34742-3 (PMC9674652; doi:10.1038/s41467-022-34742-3)
Supplement: Supplementary file 3 — Description of additional supplementary files [file 41467_2022_34742_MOESM3_ESM.pdf]

**Description of Additional Supplementary Files**

File Name: Supplementary Data 1
Description: Table listing the impact on aggregation rates for Aß42 variants for which kinetic measurements could be retrieved from the literature and the qualitative agreement or disagreement with our assay.

File Name: Supplementary Data 2
Description: List of candidate fAD pathogenic variants with increased nucleation (FDR=0.1).

File Name: Supplementary Data 3
Description: Processed data required to reproduce the analysis and figures in this paper, with read counts, nucleation scores, FDR category, associated error terms and associated pathogenicity.

File Name: Supplementary Data 4
Description: List of N-terminal Aß truncations reported in the literature and their corresponding nucleation score and category.

File Name: Supplementary Data 5
Description: List of oligonucleotides used in this study.
