# Supplementary material for: An atlas of amyloid aggregation: the impact of substitutions, insertions, deletions and truncations on amyloid beta fibril nucleation
Source: Nat Commun. 2022 Nov 18;13:7084. doi: 10.1038/s41467-022-34742-3 (PMC9674652; doi:10.1038/s41467-022-34742-3)
Supplement: Supplementary file 9 — Reporting Summary [file 41467_2022_34742_MOESM9_ESM.pdf]

nature portfolio

Corresponding author(s): Benedetta Bolognesi, Ben Lehner

Last updated by author(s): Nov 2, 2022

Reporting Summary

Nature Portfolio wishes to improve the reproducibility of the work that we publish. This form provides structure for consistency and transparency in reporting. For further information on Nature Portfolio policies, see our Editorial Policies and the Editorial Policy Checklist.

Statistics

For all statistical analyses, confirm that the following items are present in the figure legend, table legend, main text, or Methods section.

n/a	Confirmed	
☐	☒	The exact sample size (*n*) for each experimental group/condition, given as a discrete number and unit of measurement
☐	☒	A statement on whether measurements were taken from distinct samples or whether the same sample was measured repeatedly
☐	☒	The statistical test(s) used AND whether they are one- or two-sided<br>*Only common tests should be described solely by name; describe more complex techniques in the Methods section.*
☐	☒	A description of all covariates tested
☐	☒	A description of any assumptions or corrections, such as tests of normality and adjustment for multiple comparisons
☐	☒	A full description of the statistical parameters including central tendency (e.g. means) or other basic estimates (e.g. regression coefficient) AND variation (e.g. standard deviation) or associated estimates of uncertainty (e.g. confidence intervals)
☐	☒	For null hypothesis testing, the test statistic (e.g. *F*, *t*, *r*) with confidence intervals, effect sizes, degrees of freedom and *P* value noted<br>*Give P values as exact values whenever suitable.*
☒	☐	For Bayesian analysis, information on the choice of priors and Markov chain Monte Carlo settings
☒	☐	For hierarchical and complex designs, identification of the appropriate level for tests and full reporting of outcomes
☐	☒	Estimates of effect sizes (e.g. Cohen's *d*, Pearson's *r*), indicating how they were calculated

Our web collection on statistics for biologists contains articles on many of the points above.

Software and code

Policy information about availability of computer code

Data collection	No software was used for data collection.
Data analysis	All software code and custom scripts are available on GitHub: https://github.com/lehner-lab/DiMSum for raw read processing and https://github.com/BEBlab/DIM-abeta for all downstream analysis and to produce all figures, with R v3.6.3. ROC curves and AUC values were built and obtained using the 'pROC' R package (v1.17.0.1).

For manuscripts utilizing custom algorithms or software that are central to the research but not yet described in published literature, software must be made available to editors and reviewers. We strongly encourage code deposition in a community repository (e.g. GitHub). See the Nature Portfolio guidelines for submitting code & software for further information.

Data

Policy information about availability of data

All manuscripts must include a data availability statement. This statement should provide the following information, where applicable:

- Accession codes, unique identifiers, or web links for publicly available datasets
- A description of any restrictions on data availability
- For clinical datasets or third party data, please ensure that the statement adheres to our policy

Raw sequencing data and the processed data table are deposited in NCBI's Gene Expression Omnibus (GEO) as GSE193837 [https://www.ncbi.nlm.nih.gov/geo/query/acc.cgi?acc=GSE193837]. The processed data are provided in the Supplementary Data 5. Source data are provided with this paper. The coordinates for the PDB structures were obtained with accession: 7Q4M [https://doi.org/10.2210/pdb7Q4M/pdb] and 7Q4B [https://doi.org/10.2210/pdb7Q4B/pdb].

Field-specific reporting

Please select the one below that is the best fit for your research. If you are not sure, read the appropriate sections before making your selection.

[x] Life sciences [ ] Behavioural & social sciences [ ] Ecological, evolutionary & environmental sciences

For a reference copy of the document with all sections, see nature.com/documents/nr-reporting-summary-flat.pdf

Life sciences study design

All studies must disclose on these points even when the disclosure is negative.

Sample size	Efficient transformation and large enough population sizes ensured that each variant in the population (n=3164) was expected to be represented at least 10x at each step in the experiments and library preparation. For plasmid library construction, a total of 50,000 transformants were estimated, covering >15 each variant in the library. For yeast transformation, a total of 118,125, 152,000 and 139,500 transformants were estimated for each biological replicate respectively, covering >37 times each variant in the population. In vivo selection assays were performed in five technical replicates for each of 3 biological replicates, which ensured reduced noise due to batch effects.
Data exclusions	Sequencing reads that did not pass the QC filters using DiMSum package were excluded (https://github.com/lehner-lab/DiMSum).
Replication	Selection experiments were performed in 3 independent biological replicates with 5 technical replicates each (more details in the methods section). All attempts at replication were successful.
Randomization	Not relevant for this study. All environmental conditions were the same for all biological and technical replicates.
Blinding	Not relevant for this study. This work did not include cases and intervention categories.

Reporting for specific materials, systems and methods

We require information from authors about some types of materials, experimental systems and methods used in many studies. Here, indicate whether each material, system or method listed is relevant to your study. If you are not sure if a list item applies to your research, read the appropriate section before selecting a response.

Materials & experimental systems

n/a	Involved in the study	
[x]	[ ]	Antibodies
[x]	[ ]	Eukaryotic cell lines
[x]	[ ]	Palaeontology and archaeology
[x]	[ ]	Animals and other organisms
[x]	[ ]	Human research participants
[x]	[ ]	Clinical data
[x]	[ ]	Dual use research of concern

Methods

n/a	Involved in the study	
[x]	[ ]	ChIP-seq
[x]	[ ]	Flow cytometry
[x]	[ ]	MRI-based neuroimaging
